# A passive hutch-cooling system for achieving high thermal-stability operation at the Nanoprobe beamline, Diamond Light Source

**DOI:** 10.1107/S1600577520004932

**Published:** 2020-05-19

**Authors:** Fernando Cacho-Nerin, Julia E. Parker, Paul D. Quinn

**Affiliations:** a Diamond Light Source Ltd, Harwell Science and Innovation Campus, Didcot, Oxfordshire OX11 0DE, UK

**Keywords:** nanoscale beams, thermal stability, radiant panels, X-ray hutch design

## Abstract

The development of low-emittance storage rings and the rapid developments in nano-optics and imaging techniques are leading to decreasing X-ray spot sizes and increasing requirements on the environmental and mechanical stability of beamline components. In particular, temperature stability in the experimental hutches is critical to minimize uncontrolled displacements caused by thermal expansion and ensure consistent performance. Here, the design and thermal performance of the experimental hutches of the Nanoprobe beamline at Diamond Light Source are described, where a standard deviation of the room temperature down to 0.017°C over extended periods is demonstrated. The rooms are kept at constant temperature using water-cooled radiant panels which line the ceiling and walls. Radiant panels are relatively common in high-end electron microscopy rooms, but this is the first demonstration of their use for fine temperature control in an X-ray hutch and may provide a useful basis for future upgrades at upcoming low-emittance sources.

## Abstract 2

A novel strategy to achieve very high temperature stability in X-ray hutches using radiant panels is described. At maximum heat load, a standard deviation down to 0.017°C over 15 h and drift well below 0.001°C h^−1^ are demonstrated. After a sample change, the room recovers stability within 15 min.

## 1. Introduction   

Scanning X-ray microscopy is a widely implemented technique at synchrotrons worldwide because of its potential for high spatial resolution and its versatility to probe physical and chemical properties of the sample simultaneously. Continuous technological advances have pushed the beam size well below the micrometre range, far surpassing the spatial resolution of advanced optical-microscopy techniques. In the hard X-ray regime, the term ‘nanoprobe’ has been coined to refer to beamlines where the beam size is significantly smaller than half a micrometre. Several such facilities exist worldwide and more are being built or designed to meet the strong demand for these instruments. Their performance will be enhanced with the advent of diffraction-limited storage rings featuring smaller sources, higher brilliance and a larger coherent fraction.

Considering their focusing technology, nanoprobes can be divided into two broad categories: those using diffractive optics and those using Kirkpatrick–Baez (KB) mirrors. The former include Fresnel zone plates, implemented at P06 at Petra III/DESY (Schroer *et al.*, 2010 ▸), Nanoscopium at Soleil (Somogyi *et al.*, 2010 ▸), OMNY/cSAXS at the SLS (Holler *et al.*, 2014 ▸) and the Nanoprobe at the APS (Winarski *et al.*, 2012 ▸); and multilayer Laue lenses, used at the HXN beamline at the NSLS II (Yan *et al.*, 2013 ▸; Yan, Huang *et al.*, 2019 ▸). KB mirrors, on the other hand, are used at ID16 at the ESRF (Martínez-Criado *et al.*, 2016 ▸), NanoMAX at MAX IV (Johansson *et al.*, 2013 ▸), Nanoscopium at Soleil (Somogyi *et al.*, 2010 ▸), the Nanoprobe beamline at the Taiwan Photon Source (Chang *et al.*, 2013 ▸), beamline BL37XU at SPring-8 (Mimura *et al.*, 2007 ▸) and the Carnaúba beamline at Sirius/LNLS (Tolentino *et al.*, 2017 ▸), as well as the Nanoprobe beamline at Diamond Light Source (Quinn *et al.*, 2018 ▸).

In terms of technological development, the main obstacle to achieving higher spatial resolution remains the difficulty of fabricating nano-focusing optics (Ice *et al.*, 2011 ▸), be it zone plates, multi-layer Laue lenses, silicon compound refractive lenses or KB mirrors. As all these technologies currently enable beam sizes of 30 nm or less, the choice is dictated by other aspects, such as the balance between focused spot size and focal length of the optic. Diffractive optics achieve the smallest probes but offer comparatively low efficiency, are chromatic and typically offer short working distances. On the other hand, reflective optics are achromatic and boast very high efficiency with working distances of several centimetres, which become important for spectroscopy applications and *in situ* experiments, but achieve comparatively large spot sizes. The Nanoprobe beamline at Diamond uses a KB system for achromatic focusing because of its strong initial focus on spectroscopy applications. The long focal length was especially desirable to protect the costly optic, which is housed in an independent chamber under different atmospheric conditions than the sample (*e.g.* vacuum *versus* ambient pressure).

While a fundamental metric of nanoprobes, the spot size is not the only aspect determining resolution. Scanning probe techniques require rastering the sample in order to build an image and therefore the maximum achievable resolution is limited not only by the probe size but also by the scan step size, whichever is larger. As the probe size decreases, environmental effects such as vibrations and thermal fluctuations become a significant factor limiting the practical resolution of the instrument. A fundamental assumption of the image-building process is that the spot size and position of the incident X-rays do not change during the scan, at least not significantly with respect to the spot size. In other words, only the sample moves. This imposes tight positioning tolerances on the focusing optics. For reference, the KB mirrors required for achromatic nano-focused beams in the hard X-ray range typically perform optimally within ±50–100 nrad of their design incidence angle. Similarly, while less sensitive to angular changes, the position of the focal spot produced by diffractive optics translates with the optic. Therefore, a stable environment becomes a prerequisite for high resolution.

In clean rooms and high-end metrology rooms, very precise air temperature control is achieved by having rapid airflow at fixed temperatures. This strategy has been applied successfully in at least one beamline after some iteration (Martínez-Criado *et al.*, 2016 ▸; Martínez-Criado, personal communication). Here we describe an alternative approach of increasing the thermal mass of the room and reducing the airflow using radiant cooling, following design principles from the field of electron microscopy (Roulet *et al.*, 1999 ▸; Dempster *et al.*, 2001 ▸; Muller *et al.*, 2006 ▸). We discuss the design of the experimental hutches of the hard X-ray Nanoprobe at Diamond Light Source, with a particular focus on its thermal performance. We start with a brief discussion on the stability requirements, followed by a description of structural aspects including vibration, our solution to achieve high thermal stability, and results of the hutches at maximum heat load as well as during normal operation.

## 2. Stability requirements   

The concept of stability is well known and revolves around the idea of unintended or uncontrolled change in a system, or equivalently its susceptibility to perturbation. This leads immediately to two complementary considerations about the magnitude of the change and the dynamic response of the system to perturbation. The maximum allowed magnitude of the change defines the tolerance to consider the system ‘stable’; in contrast, the dynamic response of the system determines its sensitivity to excitation.

In the case of X-ray nanoprobe hutches, our concern is temperature and motion stability because of their direct impact on experiment data quality. The associated problems can be broadly grouped into low- and high-frequency issues, and their impact with regard to sample/image drift and image distortion or noise depends on the experiment and noise source, respectively. Uncontrolled motion (vibration) is typically in the 15–200 Hz range and can present itself as pixel or row distortion in scanning-probe experiments. Slow drift caused by thermal fluctuations happens over longer time scales and typically induces intensity or image drift (translation or shearing of the image).

A practical criterion to define the stability requirement of a hutch is the so-called 10% rule: the maximum allowed positional uncertainty in the instrument is 10% of the beam size. This tolerance band is applied over a time window that is intrinsically experiment dependent. However, it is unquestionable that as the beam size decreases the difficulty to maintain stability over extended periods increases. The design specification for the Nanoprobe beamline at Diamond is 50 nm focus and thus a root mean square (RMS) error of only 5 nm is desired. It must be stressed that this number refers to the uncertainty in the beam position on the sample and therefore captures both the positional stability of the focusing optic and the sample, as well as drift caused by thermal fluctuation. While sample drift can easily be corrected by appropriate post processing of the data (provided actual positions are logged and reliable), positional and angular changes in the focusing optic affects the position and size of the focal spot on the sample. A similar challenge has been faced and tackled by the electron-microscopy community in the push for high-resolution performance over long periods. Mechanical effects such as thermal drift, air currents and pressure fluctuations all have a measurable effect on the microscope, which requires a specially designed room to achieve optimal performance (Muller & Grazul, 2001 ▸; Soueid *et al.*, 2005 ▸; Muller *et al.*, 2006 ▸; Smith, 2008 ▸; Martínez-Tejada, 2014 ▸).

Significant effort has been dedicated to understanding and minimizing sources of vibration to optimize the structural behaviour of nanoprobe hutches (Simos *et al.*, 2008 ▸, 2019 ▸; Kearney *et al.*, 2019 ▸; Simos, 2019 ▸). However, although temperature stability is mentioned in every design, little detail is available about the solution finally adopted or its performance. The typical value quoted for high-stability hutches is a band of ±0.1°C around the nominal temperature (Maser *et al.*, 2014 ▸; Johansson *et al.*, 2013 ▸), compared with the usual ±0.5 to 1°C of traditional beamlines. Air-handling units are commonly deployed to handle temperature/climate control in X-ray hutches. These can achieve remarkable air-temperature stability bands (Yan, Tsai *et al.*, 2019 ▸), at the expense of an involved design and customized ducting that may not be appropriate for frequent access. For operating hutches requiring improvement, careful analysis can help inform possible lines of intervention (Baker *et al.*, 2010 ▸).

The previous discussion implicitly assumes a stationary system. In this condition, perturbations are small by design and can be attributed mainly to background vibration, to imperfections in the motion hardware, and to thermal fluctuations from the environment and from non-constant but unavoidable heat sources, *e.g.* stepper motors. Inevitably, the conditions outside the hutch are different and subject to much less stringent controls. Therefore, an important question is how long it takes for the hutch to reach equilibrium after a major perturbation such as a sample change, since it has potential impact on the time effectively available for experiments at the highest resolution.

Experimental hutches at Diamond, and elsewhere, are typically designed with a ±0.5°C acceptable temperature variation. At a nominal beam height of 1.4 m from the floor, a step change of 1°C would induce a positional change of 16 µm in the vertical direction, assuming a 1 m block of granite and 400 mm of aluminium and steel (Baker *et al.*, 2010 ▸), but in practice the actual drift will depend on the relative location of the optics and sample. More importantly, this drift would happen over a period of time τ that depends on the density ρ and specific heat capacity *C_p_* of the materials involved, their surface-to-volume ratio *A/V*, and the film coefficient *h* (to exchange heat convectively with the environment),
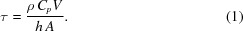



Under the effect of natural convection, a step change of 1°C (from minimum to maximum acceptable temperatures in the nominal band) would require about 5–6 h to reach a stable configuration, while with forced air flow the time would be ∼1–2 h (Baker *et al.*, 2010 ▸). Note that the positional drift in this example is from the floor to the sample position; objects at a similar height mounted on the same granite base (*e.g.* focusing optics) would drift according to the corresponding material combination and thus the relative displacement would be significantly smaller but equally unpredictable. This behaviour and the time for stabilization should ideally be mitigated for experiments using nano-focused beams. Since typical engineering materials such as steel and aluminium exhibit high coefficients of thermal expansion (CTE), while low CTE materials have poor engineering properties (see Table 1 ▸), avoiding drift in practice involves reducing thermal fluctuations as much as possible or changing the time scales over which they occur.

## 3. Structural design of the hutch   

A fundamental issue for thermal and mechanical stability is structural design. The experimental hutches of the Nanoprobe beamline are located ∼100 m away from the synchrotron building, in a satellite building which also hosts an electron microscopy facility. Thus, the behaviour of both the floor and the walls and ceiling of the hutches were considered with care, both in isolation and with respect to the rest of the building. Because they are structurally independent, the main concern during the design phase was to minimize the relative displacement between the storage ring and the endstation in the experimental hutch. This was achieved by building the latter on an 800 mm-thick floor slab detached from the soil below and supported on a 14 m-deep pile foundation. This construction mirrors the floor structure of the Diamond storage ring (Walker, 2003 ▸) and ensures that seasonal changes caused by the water table affect both buildings similarly (Kay *et al.*, 2011 ▸).

**Figure 1:**
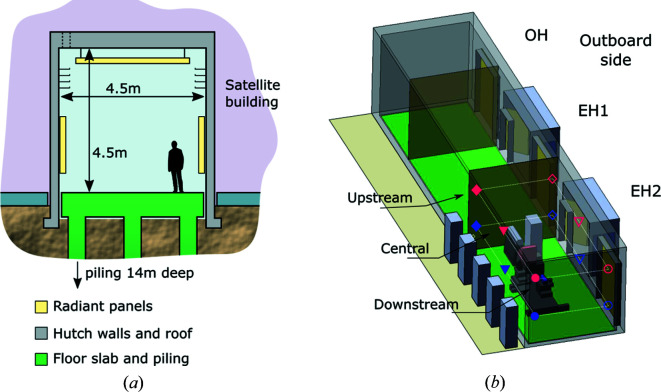
Schematic representation of (*a*) the main structural features of the hutches in the satellite building and (*b*) the space distribution within the concrete walls, see the main text for details. (*a*) The thick floor slab is supported on piles and does not touch the soil. The walls and ceiling are made of reinforced concrete and rest on their own independent foundations. This construction serves as both thermal insulation and a radiation shield. Wall and ceiling penetrations are minimized and protected by labyrinths. Cables and pipes are installed at height and run behind the radiant panels, which cover as much surface as possible of the walls and ceiling and can swing on hinges to provide access. (*b*) OH, optics hutch; EH*x*, experimental hutches. The symbols in EH2 indicate the approximate positions of the temperature sensors used to perform the measurements described here, while the colours and shapes match those used in subsequent figures.

As depicted in Fig. 1 ▸(*a*), the 300 mm-thick walls of the hutch are made of reinforced concrete and rest on their own foundations, which are independent of the piles and the rest of the building. There is a gap of 50 mm between the hutch floor and the walls, and between the walls and the building floor. The 300 mm-thick roof is monolithic with the walls. Concrete is very well suited as a construction material for high-stability environments because of its low thermal conductivity and good vibration-damping properties. The use of concrete makes these hutches very different to those of other hard X-ray beamlines at Diamond, and elsewhere, which are typically made of lead and steel plate. Here the thick hutch walls and ceiling provide uniform thermal insulation, structural support for all installations including a 2 T crane, and radiation shielding in the photon-energy range of the beamline (up to 23 keV).

The behaviour of the experimental hutches in terms of ground vibration was measured at day and night with accelerometers located on the floor of both the storage ring and the external building. The data reveal that the hutch behaviour is very similar to that of the storage ring (see Fig. S1 in the supporting information). Both spectra are dominated by low-frequency vibrations, which are most sensitive to acoustic excitation. As shown in Table 2 ▸, in both cases there is an increase in the RMS magnitude of the vibration during daytime, which we attribute to the higher activity during these hours. The similarity is important because all beam-defining optical elements of the beamline are located close to the storage ring, within optics hutches in the main synchrotron building. These currently include two harmonic rejection mirrors, a monochromator and a secondary source aperture (Quinn *et al.*, 2018 ▸).

As shown schematically in Fig. 1 ▸(*b*), the enclosure defined by the walls and ceiling is partitioned along the beam direction into three spaces by thick steel plate, supported from the walls and ceiling but not touching the floor. The first of these is a relatively small hutch dedicated to optics and diagnostics (OH), while the other two are equally sized experimental hutches (EH). The thickness of the dividing walls is designed to act as radiation shielding, so that using the beam in one hutch does not preclude entering the other. The nominal temperature in these spaces is the same but each one is regulated and balanced independently.

In terms of access, each hutch features a large cargo door to bring in large equipment, which is closed in normal operation. Regular entrance to the experimental hutches is through a smaller single-person lead door, which is accessed through a vestibule to minimize air exchange with the external corridor and reduce temperature drift during sample changes. The hutch design minimizes the number of penetrations in the walls, which are limited to these doors, a small labyrinth at low height in each hutch for user experiments and the necessary opening for the beam pipe. All fixed installations enter the hutches through openings in the roof, also protected by labyrinths. These smaller features have been omitted in Fig. 1 ▸(*b*) for clarity.

## 4. Radiant panels   

Radiant panels are specially designed heat-exchange elements that emphasize radiation over convection and, as a result, the surrounding environment features less natural air circulation. Although these systems have been deployed successfully in commercial and office environments since the mid-1900s (Giesecke, 1946 ▸; Manley, 1954 ▸), radiant systems are less popular than all-air or conventional radiator systems and, as far as the authors are aware, they have never been used as a primary cooling system in synchrotron experimental hutches.

**Figure 2:**
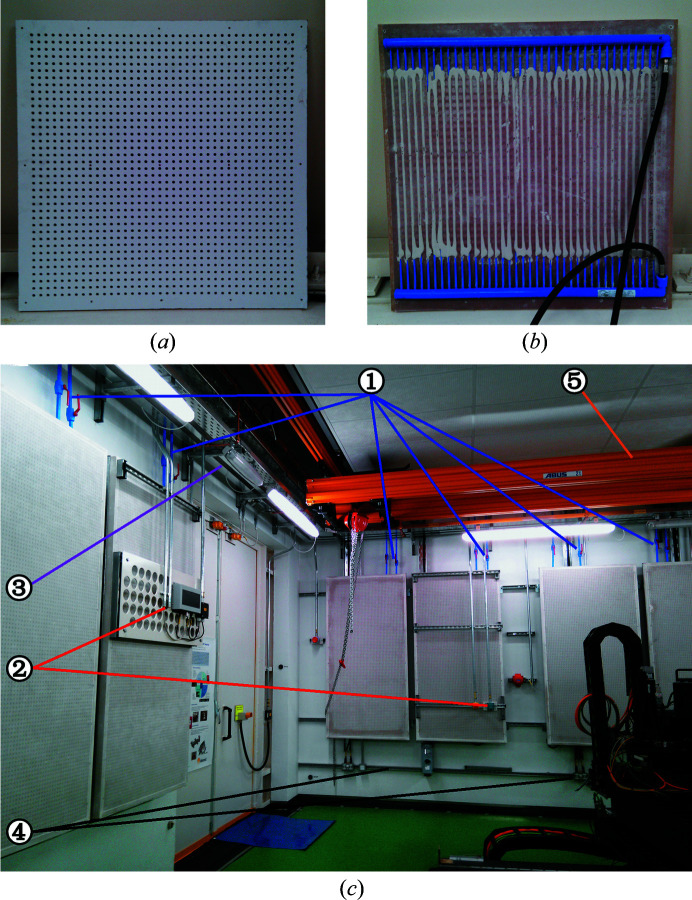
Radiant panels installed in the Nanoprobe beamline, see the main text for details. (*a*) Front and (*b*) back of an individual tile, before sealing. (*c*) An experimental hutch where the main features of the installation can be seen: panel pipework [(1), blue lines], wall-mounted elements [(2), red lines], high-level electrical containment [(3), purple lines] and low-height installations [(4), black lines] as well as the crane and ceiling-mounted panels [(5), orange lines]. The panels on the walls are assembled on steel frames which can rotate on hinges for access.

The radiant panel elements used in the nanoprobe are pre-formed water-cooled plaster tiles, as shown in Fig. 2 ▸. This is very different from typical solutions deployed for electron microscopy, which are usually made of metal. The porous nature of plaster greatly increases the exposed surface, aiding radiation even at room temperature. The tiles are perforated with a pattern of circular holes, which increases their total surface, makes them lighter and has soundproofing properties. The water circulates through capillary tubing embedded in the gypsum. On the side exposed to the hutch, a thin glass fibre membrane prevents dust shedding and air circulation through the holes, further inhibiting natural convection and improving the soundproofing qualities to Class C (highly absorbing). The back side of the panel is insulated and sealed to make sure that heat is only exchanged through the front. Radiation is estimated to account for 60% of the heat-exchange performance of the panels, with the rest caused by natural convection. The cooling power depends on the temperature difference between the ambient and the cooling water, Δ*T*, as reported in Table 3 ▸.

The radiant panels lining the ceiling are mounted on a lightweight support structure similar to office environments. By design the exposed panel surface is at the same level as the crane rails [see Fig. 1 ▸(*a*)], such that there is no apparent loss of headroom. All necessary installations, in particular the pipework, run between the panels and the ceiling and are thus hidden from view.

For the walls, an important constraint was that the underlying surface remained available for future installations, *e.g.* to lay additional pipework or cabling. This is a major difference with respect to typical transmission electron microscopy rooms where the wall is permanently concealed. To fulfil this requirement, we designed steel frames with hinges that allow swinging the panel like a cupboard door. With this system, all cable and pipe runs can be fixed to the wall and covered by the radiant panels. This construction greatly increases the effective exposed surface and thus the overall performance, while preserving a tidy appearance. Wall-mounted elements that must be visible and/or accessible, such as radiation protection alarms and notification elements, are installed on perforated plates and affixed to the steel frame. This reduces the available surface area minimally. All other wall-mounted elements, such as power and network sockets, are installed at low height using a rail system on the concrete wall. This minimized the total number of holes drilled into the wall and thus the possible sources of dust inside the hutch.

Dust is a general concern at beamlines using scintillator-based detectors as the intense ionizing radiation can cause electrostatic charge build up. As a result, dust particles are attracted to the screen, degrading the image in a non-systematic manner and in some cases appearing as over-saturated spots which cannot be flat-field corrected. At the Diamond Nanoprobe, dust is an important concern as the sample is usually placed in an open vessel under ambient conditions while the focusing KB mirror is under ultra-high vacuum. Therefore, dust can settle on the thin window separating these two spaces, as well as on the optical elements of the interferometry setup controlling the sample position. An additional concern in the design phase was the risk of sample contamination. While the user community of the beamline covers a wide range of scientific fields, their samples are typically a few micrometres in size, which is comparable with a typical grain of dust and therefore the risk of contamination cannot be neglected in the design. In this sense, the lack of air motion afforded by the radiant panels is an important element in keeping the hutch clean without imposing an unnecessary burden on its users.

### 4.1. Temperature regulation   

By design, the radiant panels described above act as an inertial system, in the sense that they respond slowly to perturbations and they dampen rapid changes very effectively. This is because of the high heat capacity of water and the large total mass flow.

The lowest safe operating temperature of the cooling water is determined by the dew point to avoid condensation in the hutch. In turn, the dew point depends on the air temperature and humidity. To break this circular dependency, the cooling-water temperature is set to 17°C, which is above the dew point for all conditions of air temperature up to 21°C and relative humidity up to 70%. The nominal relative humidity for the hutch is 40% ± 5%.

For a given heat load, it is clear that variations in the temperature of the cooling water lead to variations in the stable air temperature within the hutch. Conversely, variations in the heat load result in a different equilibrium temperature even for constant coolant flow conditions. Therefore, the key to obtaining optimal performance (defined by the stability during operation) is to keep the inlet water temperature as constant as possible, while simultaneously reducing variations in the heat load.

Because of the inertial character of radiant cooling, changes to the coolant temperature are slow to propagate into the room. This makes it a challenge to prescribe and track a stable temperature in an environment where the heat load is mostly constant but subject to slow static drift and to occasional short-term spikes caused by, for example, scientists entering the room to change samples. This combination of fast and very slow cycles can lead to oscillations in typical control loops. Instead, keeping the coolant temperature constant and letting the room return to its natural equilibrium is straightforward. This strategy has therefore been adopted, accepting its inherent inability to prescribe the stable temperature in the room directly.

**Figure 3:**
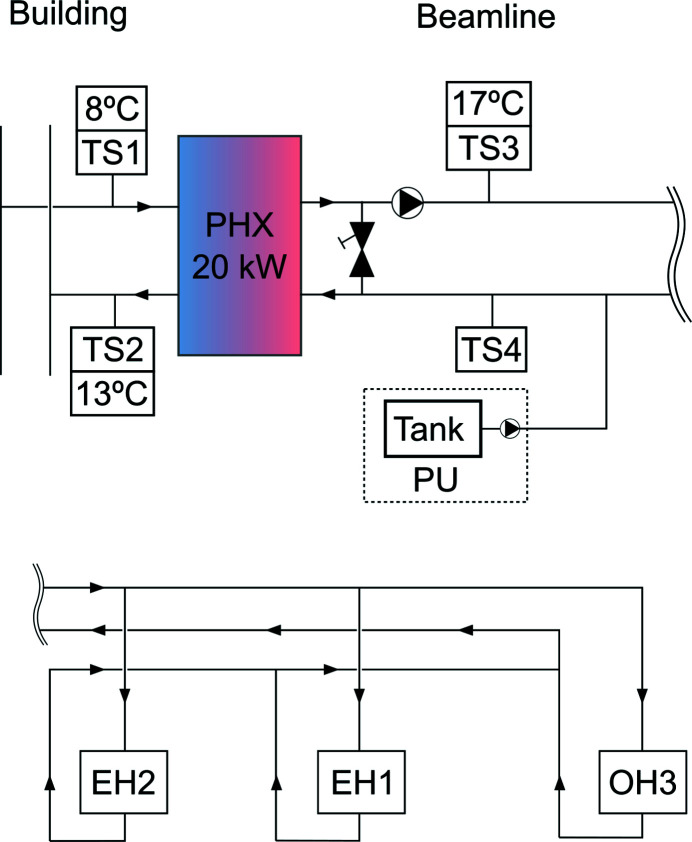
A schematic view of the cooling circuit design for the radiant panels, showing the main elements. These include: TS*x*, temperature sensors; PHX, plate heat exchanger; PU, pressurization unit; EH*x*, experimental hutches; and OH3, optics hutch 3. See the main text for a detailed explanation.

For the cooling water flowing through the radiant panels a stability band of ±0.1°C was prescribed around the setpoint of the inlet cooling water. This performance specification is in line with that of high-end metrology and electron microscopy environments. In order to achieve this performance level using the main building process cooling water, the installation is designed as a chain of water-cooled mixing circuits as depicted in Fig. 3 ▸. This design strategy has several advantages: (i) Because the circuits are independent, water quality on the radiant panel side does not degrade. (ii) Mass flow on the radiant panel side is constant so no tuning is required after commissioning. (iii) The pipework internal to the hutch, including the capillary tubing, is made of polypropyl­ene, which is slightly oxygen permeable. Having independent circuits allowed us to worry about pipe-material compatibility and rust only in the radiant panel circuit, without affecting the rest of the building infrastructure. (iv) The temperature of the plant cooling water may be variable (±1°C as per building specification) and have a different setpoint without affecting radiant-panel performance. (v) Using the main plant process water makes the system more space and energy efficient, and simpler to manage at facility level.

A plate heat exchanger (PHX in Fig. 3 ▸) separates the building from the radiant panel circuits. On the beamline side, a mixing bypass controlled by a variable flow valve allows fine control of the temperature flowing into the radiant panels. The mixing ratio is controlled by the temperature of the building chilled water before and after the PHX (TS1 and TS2), and by the flow and return temperature of the radiant panel water (TS3 and TS4). An autonomous pressurization system (PU) keeps pressure constant and compensates for small losses, *e.g.* as a result of maintenance.

The pipework layout is designed as an equal-length system throughout, so that the pressure loss is the same in all three hutches. Outside the hutches this is achieved by adapting the pipe diameters at branching points, and by arranging the inlets and outlets of the hutches in first-in/last-out order. This is depicted schematically in Fig. 3 ▸. Within the hutches, the surface covered by radiant panels was divided into zones of roughly the same area in order to have a uniform pressure drop in all branches, while adopting the same first-in/last-out principle for the flow and return legs. This design is self-equilibrating and ensures uniform flow across all branches of the system.

### 4.2. Fresh air supply   

Although experimental and optics hutches remain closed while in operation, it is required that there is a supply of fresh air at all times. The fresh air supply also helps to control relative humidity and builds up a positive atmospheric pressure that prevents dust from entering the room, but works against our temperature stability goals which effectively require that air currents be minimized. These include not only natural convection from warm surfaces, but also eddies arising from air mixing as it enters the room. Moreover, it is clear that a temperature mismatch between the hutch air and any fresh intake immediately results in a perceived load on the radiant panel system. Local heat loads are kept constant in the hutch and therefore the main source of perceived instability in our hutches is air currents and draughts. In an enclosed space, these arise naturally from the gradients caused by slight temperature differences at different points, as well as from natural convection from unavoidable heat sources. As explained above, our radiant panels are engineered to minimize convection as much as possible and work close to the target temperature of the hutch, reducing air motion near their surface.

In order to eliminate eddies from the fresh air supply, a textile diffuser has been installed to distribute the outlet over a large area and ensure slow laminar flow. Additionally, fresh air enters the hutch at the equilibrium temperature, eliminating eddies caused by mismatch. This was achieved by turning off the fresh air supply and restricting access over a 60 h time period, such that the hutch could reach and maintain equilibrium. The temperature of the fresh air supply was then set to this equilibrium temperature (to the nearest 0.1°C).

Therefore, the air conditioning is used only to provide fresh air at a precise temperature and very low flow. This is very different to other beamlines at Diamond, and elsewhere, where it functions as the main temperature-regulation system.

## 5. Results and discussion   

The temperature of the hutches is monitored via 12 sensors installed ∼1 m from the ground (referred to as ‘low’ hereafter) and close to the ceiling (‘high’) in all corners of the hutch, as well as at the sample position, *i.e.* mid-length of the hutch along the beam propagation direction. This is depicted schematically in Fig. 1 ▸(*b*). The sensors are a mixture of K-type thermocouples (in the corners) and Pt-100 sensors (in four-wire configuration) and are all monitored from the same instrument (model PTC10, Stanford Research Systems, CA, USA). The intrinsic variability in the response of these sensors means that the exact temperature may vary by a small but significant offset from sensor to sensor. This is especially true for the thermocouples, because of the cable length, but does not affect per-sensor statistics, which is our main interest. By construction, thermocouples are also more sensitive to electronic noise picked up by the cable. This was compensated in part by placing the readout instrument in the centre of the room, such that cable length was similar for all K-type sensors. In contrast, Pt-100 sensors connected in four-wire configuration are much less sensitive to cable-related noise.

As part of the commissioning process for the radiant panel installation, a heat-load test was carried out to assess its performance. At the time there was no instrumentation in the room other than the temperature monitor, as the endstation had not been built yet. As mentioned above, the room was specified to provide a temperature stability band of ±0.1°C, which must be held over at least 2 h. The test consisted of placing heat sources (light bulbs) totalling 4 kW in the hutch, closing the doors and sealing all labyrinths to prevent heat loss through leaks. The doors were kept closed for 60 h and no access was permitted to the building or the hutches during this time. During the test the settling time of the hutches was monitored, as well as the stability achieved over a period of a few hours. Separately, the stability at low heat load over periods of time of several days was measured in order to establish long-term drift, night/day variability and time to settle after small-scale perturbations (*e.g.* entering the hutch for a few minutes).

**Figure 4:**
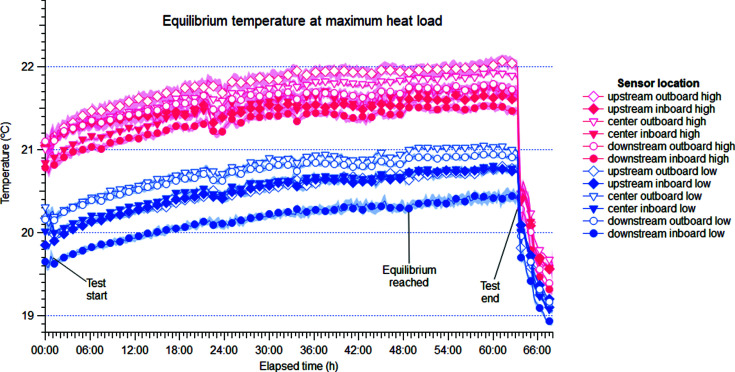
Temperature evolution in the experimental hutch during the heat-load test. Refer to Fig. 1 ▸(*b*) for a schematic representation of the sensor locations inside the hutch. For visualization purposes, the temperature logged every 10 s by each sensor has been represented as the average in 15 min intervals, with a band around the trace corresponding to the standard deviation within each interval.

Fig. 4 ▸ shows that the temperature rose steadily for 2 d during the heat-load test before stabilizing. Although this settling time may seem long, it must be noted that the perturbation to the system was as strong as possible, with an instantaneous jump in the load from 0 to 4 kW. In spite of this, rather than spiking and slowly settling down as might be expected from other cooling systems, the temperature increased smoothly.

For visualization purposes, the temperature logged every 10 s by each sensor was grouped into intervals of 15 min, for which the average and standard deviation were computed. The average values are represented in Figs. 4 ▸ and 5 ▸ with a band around each trace corresponding to the standard deviation within each interval. As mentioned above, standard deviation was higher for thermocouples than for Pt-100 sensors as the former data contain unavoidable electronic noise.

**Figure 5:**
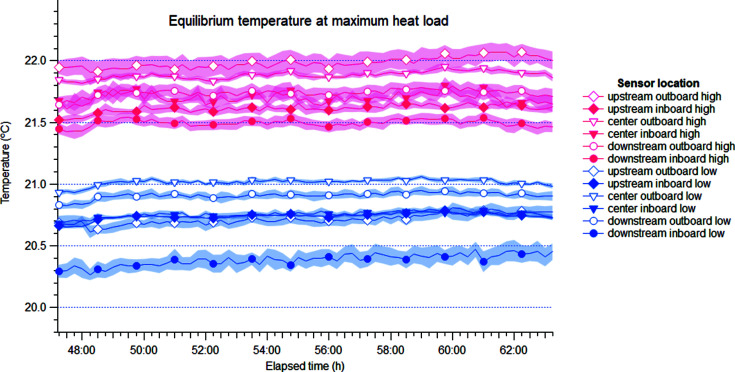
Temperature stability in the experimental hutch during the heat-load test, at equilibrium. See Fig. 1 ▸(*b*) for a schematic depiction of the sensor locations and Table 4 ▸ for statistics. Data are represented as in Fig. 4 ▸.

Once stabilized, the hutch showed good stability over the last 15 h of the test, as shown in Fig. 5 ▸. For this period, the average and standard deviation of all data points were computed (∼5400 points), revealing standard deviations ranging between 0.017°C and 0.088°C, as reported in Table 4 ▸. This is well within the specification at all sensor locations.

The strong stratification reported in Table 4 ▸ is a direct consequence of the lack of air motion within the hutch resulting from the working principle of the radiant panels. Indeed, all sensors at height report a temperature ∼1°C higher than their low counterparts, although a detailed analysis of the temperature differences cannot be carried out as these are not cross calibrated. Nevertheless, the difference between high and low sensors is significantly higher than the measurement error of the sensor, confirming that the stratification revealed by these measurements is not an artefact.

In order to establish whether the temperature drifts in the room, the last 15 h of the heat-load test was considered to reflect the steady state (Fig. 5 ▸) and a linear fit was computed for the raw data in this period (one data point every 10 s). The results are reported in Table 5 ▸ and show that stability is excellent, with slope values in the order of 1 × 10^−4^°C h^−1^. In this table, the standard deviation reported with each parameter can be interpreted as a goodness-of-fit score. In this sense, it is important to note that the uncertainty of the fit results, in particular the slope, is dominated by the intrinsic noise of the signal, which combines high-frequency oscillations from natural convection as well as electronic noise from the long cables. This is reflected by the relatively high standard deviation of the slope.

An interesting and important feature of the radiant panel system is its rapid recovery after a heat source is removed. Indeed, as seen in Fig. 4 ▸, at the end of the test the temperature returns very quickly to its original value and settles within 6 h. Small perturbations such as a single person entering the hutch do not produce a measurable change in the return water temperature in the radiant panels. The corresponding change in air temperature is also very small and can be attributed to eddies caused by the person walking in the hutch. Both of these effects are a consequence of the inertial character of radiant cooling.

Fig. 4 ▸ shows a mismatch between the hutch temperatures at the beginning and at the end of the test. This is explained by two concurring factors. First, the test was started immediately after setup was complete, without letting the room stabilize after closing the doors which had been open to the rest of the building. Second, and more importantly, the setup works involved technicians installing and testing the heat sources. These two factors effectively made the initial heat load in the room non-zero; in contrast, at the end of the test the heat load was removed completely. As explained above, the equilibrium temperature of the hutch depends on the heat load, and therefore the equilibrium temperature before and after the test is different.

In terms of the temporal evolution of the temperature, Figs. 4 ▸ and 5 ▸ show no periodic oscillations or artefacts, which are sometimes seen with PID control systems especially in the vicinity of the setpoint. This is because radiant panels remove heat from the hutch passively, reaching the natural equilibrium instead of introducing and mixing air at a lower temperature. If we consider Fig. 4 ▸ in the context of equation (1) above (*i.e.* neglecting radiation), it is easy to see that the design of the radiant panels aims to maximize the characteristic time constant of the hutch globally. The panel construction minimizes convection, effectively lowering the film coefficient *h*. The glass fibre membrane decreases the area subject to convective heat exchange, decreasing *A*. Similarly, water has a high specific heat capacity *C_p_* and the panels form a large parallel capillary network with high aggregate volumetric flow *V*. The fast cooling at the end of the test can be attributed to the combined effect of the radiant panels as the major contributor, and the mixing of cool air as a minor contribution from the fresh-air supply, which also restarted operation at this time.

**Figure 6:**
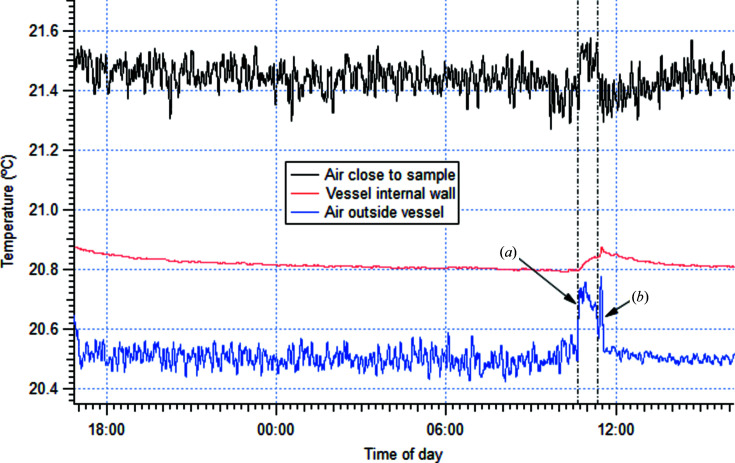
Temperature response around the sample position following a perturbation simulating a sample change. (*a*) Start of the simulation and (*b*) end of the perturbation.

While the hutch returns to its steady state very quickly after a small perturbation, it is important to quantify the impact of such perturbations in the vicinity of the sample during normal operation. In these conditions the heat load is under 1 kW, in contrast with the test reported previously. With the hutch in the steady state, the air temperature was measured above the sample vessel, as well as inside it at the sample position and on the surface of an internal vessel wall, while a single person worked around the vessel for a period of 1 h. This test simulated the effect of a sample change and lasted long enough to produce a measurable temperature variation in its immediate surrounding, as shown in Fig. 6 ▸. In order to measure the small variation expected, the sensors for this measurement were cross calibrated by clamping them to a freshly polished slab of copper, in close proximity to one another (within 20 mm). A constant offset was enough to ensure equal readings from all three probes, within the uncertainty of the instrument.

Once the sensors were installed, the hutch was closed and left to stabilize overnight to ensure the measurement captured the effect of the perturbation correctly. As shown in Fig. 6 ▸, the air temperature remains constant overnight within the noise associated with natural convection, while the vessel wall takes a few hours to reach stationary conditions. The vessel had warmed up by 0.08°C as a result of the sensor installation, which involved bolting a sensor to the wall in addition to placing the air temperature probes. We note that this temperature increment would still be within the thermal specification of the hutch, although it appears clearly out of balance in the figure. We consider this wall to be at constant temperature from ∼06:00, *i.e.* at least 4 h before the simulation started. The figure also reveals that air temperature outside the vessel is lower and slightly more stable than within. This is a direct consequence of the shielding effect of the vessel walls, which represents an obstacle to natural convection and shields thermal radiation around the sample. The temperature of the vessel wall displays much lower noise as this was a contact measurement.

Upon approaching the vessel [time point (*a*) in Fig. 6 ▸], the temperature rises suddenly by up to 0.3°C outside and 0.15°C inside the vessel. A slower and less pronounced rise is seen in the vessel wall, which can be attributed to the higher heat capacity of stainless steel. After the work was finished, the hutch was closed again [time point (*b*)] and left to equilibrate. The plot clearly shows that the air close to the outer side of the vessel recovers stability within 15 min. In close vicinity to the sample, the air temperature returns to its previous stability band in less than 30 min. The vessel inner wall takes ∼3 h to recover the previous temperature. This is consistent with the much slower warm-up process and is also expected since this is the point where heat exchange is least efficient: it happens mostly by conduction through the wall as air flow is impaired by the multitude of items inside the sample vessel.

Typical sample changes at the Nanoprobe beamline last less than 3 min and therefore the impact on the vessel-wall temperature is negligible. Our measurements show that the disturbance to the air temperature is mostly caused by eddies created by the user moving and that these are dissipated quickly. The typical amplitude of the perturbation is also small near the sample. Upon mounting a sample, the first operation a user carries out is finding an appropriate region of interest, using an optical microscope, and acquiring a coarse-resolution scan to optimize acquisition parameters. This typically takes between 15 and 30 min, *i.e.* in the same order of magnitude as the room stabilization time, and effectively removes the need for the user to wait for the hutch to stabilize.

## 6. Conclusions   

We have presented a novel strategy to achieve very high temperature stability in X-ray hutches. Our approach is to increase the thermal mass of the room using both concrete shielding and radiant panels. These water-cooled elements exchange heat with the environment primarily by radiation and create a chamber where the walls and ceiling of the cabin are kept at a constant temperature. To the best of our knowledge, this is the first time such a system has been deployed in a synchrotron hutch. The room responds slowly to perturbations and dampens rapid changes very effectively. This makes it difficult to prescribe and track an arbitrary temperature in the hutch. Our strategy is to keep the coolant temperature constant and let the room reach its natural equilibrium. This scheme is more robust and does not lead to oscillations against slow static drift. A direct consequence of our approach is that the equilibrium temperature depends on the heat load within the room and cannot be prescribed directly. We minimized heat-load variations by removing sources from the room where possible (*e.g.* motion and vacuum controllers) and ensuring that all instrumentation in the hutch stays connected at all times. Our system features minimal drift and fast recovery from perturbations, allowing experiments to start quickly and proceed uninterrupted for extended periods of time.

## Supplementary Material

Figure S1: vibration behavior of the hutch floor compared with that of the storage ring. DOI: 10.1107/S1600577520004932/fv5119sup1.pdf

## Figures and Tables

**Table 1:** Thermal and mechanical properties of typical materials used in synchrotron instrumentation

Material	Coefficent of thermal expansion α (µm m^−1^ K^−1^)	Thermal diffusivity *D* (m^2^ s^−1^)	Young’s modulus *E* (GPa)
Aluminium	23.1	9.7 × 10^−5^	69
Steel	10–17	3–4 × 10^−6^	200–210
Copper	17	1.11 × 10^−4^	117
Brass	19	4.45 × 10^−5^	100–125
Granite	8	1.13 × 10^−6^	70
Glass	3.3	3.4 × 10^−7^	50–90
Invar	1.2–1.5	2.85 × 10^−6^	137
Zerodur	0.1	7 × 10^−7^	91

**Table 2:** Vibration levels at I14 and the main synchrotron floor

Direction	Time of day	Location	RMS (nm)	PKPK (nm)
Vertical	Day	I14 floor slab	15.26	43.15
		DLS main	23.03	65.15
	Night	I14 floor slab	11.62	32.87
		DLS main	11.40	32.24
Horizontal	Day	I14 floor slab	12.94	36.59
		DLS main	16.64	47.08
	Night	I14 floor slab	11.01	31.14
		DLS main	8.292	23.45

**Table 3:** Temperature dependence of the radiant-panel cooling power

Δ*T* (°C)	Cooling power (W m^−2^)
8	62.6
10	79.2
15	86.3

**Table 4:** Stable temperatures reached under maximum heat load at different locations of the experimental hutch over the last 15 h of the test

Sensor location			Temperature average (°C)	Temperature standard deviation (°C)
Low	Upstream	Outboard	20.72	0.057
		Inboard	20.75	0.017
	Central	Outboard	21.02	0.022
		Inboard	20.76	0.029
	Downstream	Outboard	20.91	0.035
		Inboard	20.39	0.077
High	Upstream	Outboard	21.99	0.088
		Inboard	21.62	0.041
	Central	Outboard	21.89	0.035
		Inboard	21.70	0.059
	Downstream	Outboard	21.75	0.057
		Inboard	21.50	0.049

**Table 5:** Temperature drift in the experimental hutch at maximum heat load once the hutch is stable The results are from a linear fit of the temperatures over the last 15 h of test. The results are given as the coefficient value ± standard deviation of the estimate.

Sensor location			*T*_0_ (°C)	Slope (°C h^−1^)
Low	Upstream	Outboard	20.718 ± 0.0006	8.466 × 10^−4^ ± 1.411 × 10^−5^
		Inboard	20.747 ± 0.0002	2.015 × 10^−4^ ± 5.046 × 10^−6^
	Central	Outboard	21.022 ± 0.0003	5.180 × 10^−5^ ± 5.046 × 10^−6^
		Inboard	20.759 ± 0.0004	2.523 × 10^−4^ ± 8.774 × 10^−6^
	Downstream	Outboard	20.915 ± 0.0005	1.643 × 10^−4^ ± 1.083 × 10^−5^
		Inboard	20.392 ± 0.0010	7.770 × 10^−4^ ± 2.207 × 10^−5^
High	Upstream	Outboard	21.987 ± 0.0011	8.539 × 10^−4^ ± 2.549 × 10^−5^
		Inboard	21.618 ± 0.0005	3.318 × 10^−4^ ± 1.207 × 10^−5^
	Central	Outboard	21.888 ± 0.0004	4.323 × 10^−4^ ± 9.372 × 10^−6^
		Inboard	21.703 ± 0.0008	−1.613 × 10^−4^ ± 1.835 × 10^−5^
	Downstream	Outboard	21.745 ± 0.0008	9.364 × 10^−5^ ± 1.778 × 10^−5^
		Inboard	21.500 ± 0.0007	−8.777 × 10^−5^ ± 1.529 × 10^−5^
