# Supplementary material for: A passive hutch-cooling system for achieving high thermal-stability operation at the Nanoprobe beamline, Diamond Light Source
Source: J Synchrotron Radiat. 2020 May 19;27(Pt 4):912–22. doi: 10.1107/S1600577520004932 (PMC7336176; doi:10.1107/S1600577520004932)

JOURNAL OF SYNCHROTRON RADIATION

Volume 27 (2020)

Supporting information for article:

A passive hutch-cooling system for achieving high thermal-stability operation at the Nanoprobe beamline, Diamond Light Source

Fernando Cacho-Nerin, Julia E. Parker and Paul D. Quinn

Figure S1 Vibration behaviour of the floors in the storage ring and the experimental hutches (a) in the vertical and (b) horizontal direction. The general performance is equivalent, with slight differences in the low-frequency range below 20 Hz, mainly due to the different acoustic excitation. This is especially visible in the difference between night and day spectra. See Table 2 in the main text for numeric values.

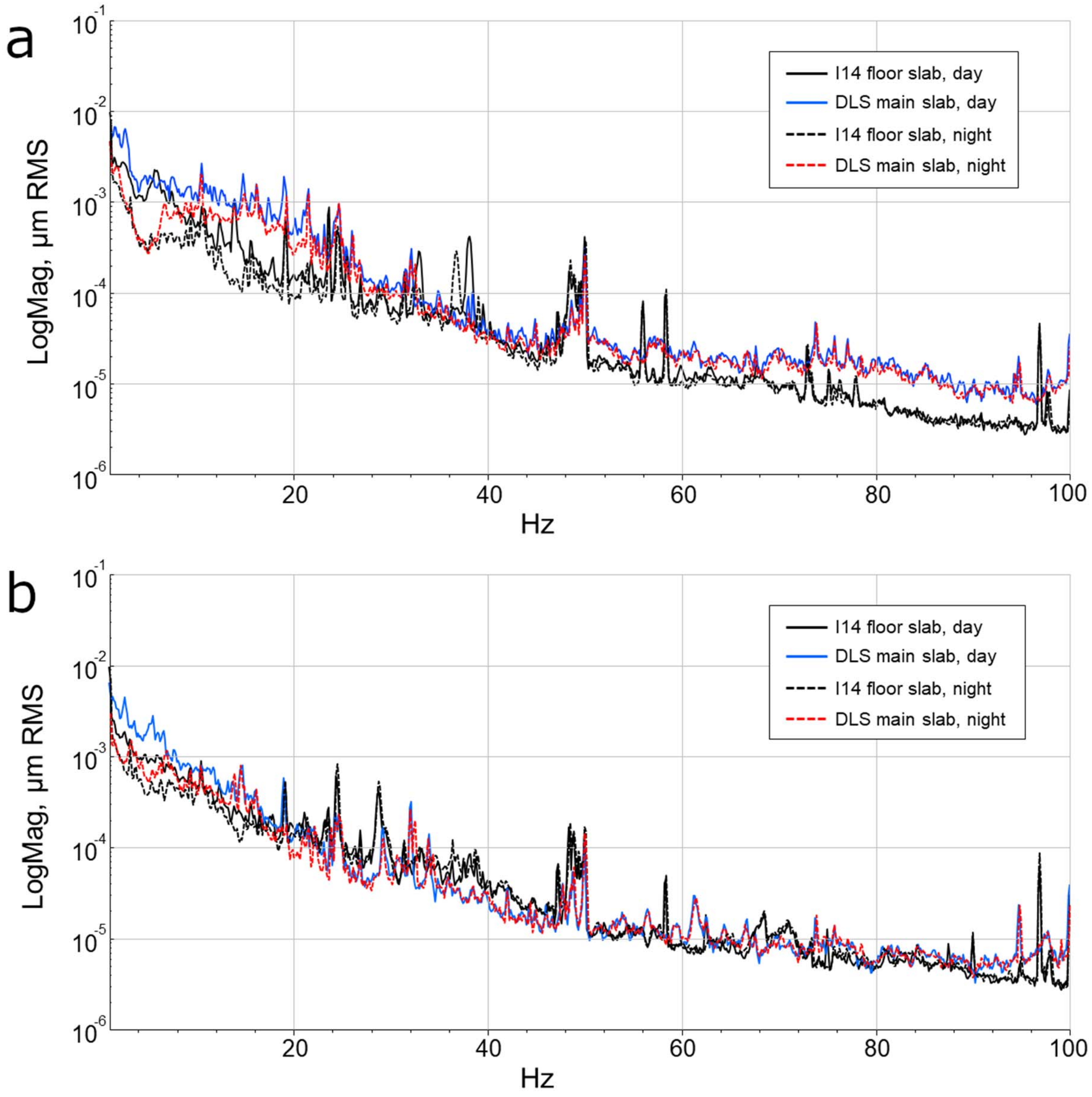

Supplement: Supplementary file 1 [file s-27-00912-sup1.pdf]
